# Supplementary material for: Chromothripsis during telomere crisis is independent of NHEJ, and consistent with a replicative origin
Source: Genome Res. 2019 May;29(5):737–49. doi: 10.1101/gr.240705.118 (PMC6499312; doi:10.1101/gr.240705.118)
Supplement: Supplemental Material [file supp_gr.240705.118_Supplemental_file_1.zip › contigs/annotated_contigs/DB73/contig.3.DB73_length_497_mean_cov_22.6720321932.docx]

**DB73_length_497_mean_cov_22.6720321932**

```
AA|TCTTAAACTTGTTCACACTTGTTTTTGTGTCCTATATGGTCTATGCTGGATCATGTTCCATGTGCACTAGAGAAGA|ATG|AGGCT
   >chr3:81208029-81208107 - E=4e-31                                           >chr3:7
TATTTGTATCTAATTTGTAACTACTCCAATTAATGTTTTTCTTAGTGAAATATATTAATGAATAATGAAAGATCTATTATTTGCTCATG
8990187-78990496 + E=2e-174 p=0e+00
TCCACCATGAAACAAAAATTATAATTTGAAATGCCAATAGTTGTATAATCTGAAACACTAATAACTTTAGCTTAGTGCACGTATACAGC

ATGGTATAGATTTTTTCAATGAAACTAAGCTCTAGAAAAGGAGGAAATAAACTAGCAATATAAAAGAAAAAAATTACTTGGAAACTTAT

ACTCTCTAAGGCTATGTTGAAGCCAAGTGAAATT|AACAAAGATATTCAGG|ATGTTAGACAGATCATTGAGACAGAAAATTAACAAAG
                                                   >chr3:80297845-80297918 + E=3e-32
ATATTCAGGACTTGAACTCAGCTCTGGACCAAGTGG|CTTGATGTTGTCCTTATAGCT
```
